# CIPHER-seq enables intracellular multimodal profiling of cytokine responses in single immune cells

**DOI:** 10.1038/s41598-026-44946-y

**Published:** 2026-04-08

**Authors:** Avni Bhalgat, Katarina Micin, Maurizio Affer, Ana C. Ayupe, Isabella Gomez, Jonathan H. Schatz, Erietta Stelekati, Sion Williams, Krishna Komanduri, Eric Wieder, Emiliano Cocco, Justin Taylor

**Affiliations:** 1 Sylvester Comprehensive Cancer Center, University of Miami Miller School of Medicine, Miami, FL USA; 2 Sheila and David Fuente Graduate Program in Cancer Biology, University of Miami Miller School of Medicine, Miami, FL USA; 3 Dr. John T MacDonald Foundation, Department of Human Genetics, Miller School of Medicine, University of Miami, Miami, FL USA; 4 Division of Hematology, Department of Medicine, Sylvester Comprehensive Cancer Center, Miami, FL USA; 5 Sylvester Onco-Genomics Shared Resource (OGSR), Sylvester Comprehensive Cancer Center, University of Miami Miller School of Medicine, Miami, FL USA; 6 Department of Microbiology and Immunology, University of Miami Miller School of Medicine, Miami, FL USA; 7 Department of Medicine, Sylvester Comprehensive Cancer Center, Miami, FL USA; 8 Division of Hematology and Oncology, Department of Medicine, University of California San Francisco, San Francisco, CA USA; 9 Helen Diller Family Comprehensive Cancer Center, University of California San Francisco, San Francisco, CA USA; 10 Sylvester Flow Cytometry Shared Resource (FCSR), Sylvester Comprehensive Cancer Center, University of Miami Miller School of Medicine, Miami, FL USA; 11 Department of Biochemistry and Molecular Biology, Miller School of Medicine, University of Miami, Miami, FL USA; 12 1011 NW 15th St, 33136 Miami, FL USA; 13 1501 NW 10th Avenue, 33137 Miami, FL USA

**Keywords:** Intracellular CITE-seq, Single-cell multiomics, Immune activation, Cytokine profiling, RNA-protein correlation, Mitochondrial stress, PBMC stimulation, Pseudotime dynamics, Proteotranscriptomics, CIPHER-seq, Biological techniques, Cell biology, Computational biology and bioinformatics

## Abstract

Single-cell RNA sequencing inadequately predicts protein abundance, particularly for intracellular cytokines. We developed CIPHER-seq (Cytokine Intracellular Protein High-throughput Expression with RNA-sequencing), enabling simultaneous quantification of intracellular proteins and transcriptomes. This system provides five layers of analysis. CIPHER-seq matched commercial protocols in immune cell recovery while significantly reducing mitochondrial stress signatures. Applied to stimulated PBMCs, CIPHER-seq resolved robust cytokine induction and metabolic remodeling, establishing a streamlined platform for intracellular multimodal profiling.

**Supplementary Information:**

The online version contains supplementary material available at 10.1038/s41598-026-44946-y.

## Introduction

Single-cell RNA sequencing (scRNA-seq) provides transcriptional resolution but fails to predict protein abundance accurately, with correlations typically ranging from *r* = 0.1–0.4 across diverse biological systems^1–4^. This discordance reflects post-transcriptional regulation, variable protein turnover, and translational control mechanisms. Proteotranscriptomic studies in various external datasets demonstrate particularly weak concordance for cytokines and activation markers^5,6^, necessitating direct protein measurement alongside transcriptomes. While surface-protein CITE-seq has enabled robust multimodal profiling^7^, and recent advances in spatial technologies have enabled tissue based mapping^8,9^, intracellular adaptation remains challenging for suspension-based populations. Standard intracellular staining protocols optimized for flow cytometry often degrade RNA or elevate mitochondrial transcripts, compromising sequencing quality^10,11^. Commercial intracellular protocols provide improvements but frequently introduce cellular stress artifacts^12,13^.

We developed CIPHER-seq, a multi-layer system integrating optimized fixation chemistry, controlled permeabilization, abbreviated antibody incubation, and RNase-protective conditions (Extended Data Fig. 1). To validate performance, we benchmarked CIPHER-seq against Proteintech, BioLegend, and BD’s intracellular protocols (Extended Data Table 1). *Tubulin*, a ubiquitously expressed cytoplasmic protein present in all nucleated cells, was used as a general indicator of intracellular antibody accessibility following fixation and permeabilization and not as a proxy for all intracellular proteins. Flow cytometry-based screening in all 4 protocols revealed that BD and BioLegend protocols failed to provide adequate intracellular antigen access for Tubulin (Extended Data Fig. 2). We therefore advanced Proteintech as the primary comparator for single-cell experiments and assessed both methods in resting and PMA/ionomycin-stimulated peripheral blood mononuclear cells (PBMCs).

**Fig. 1:**
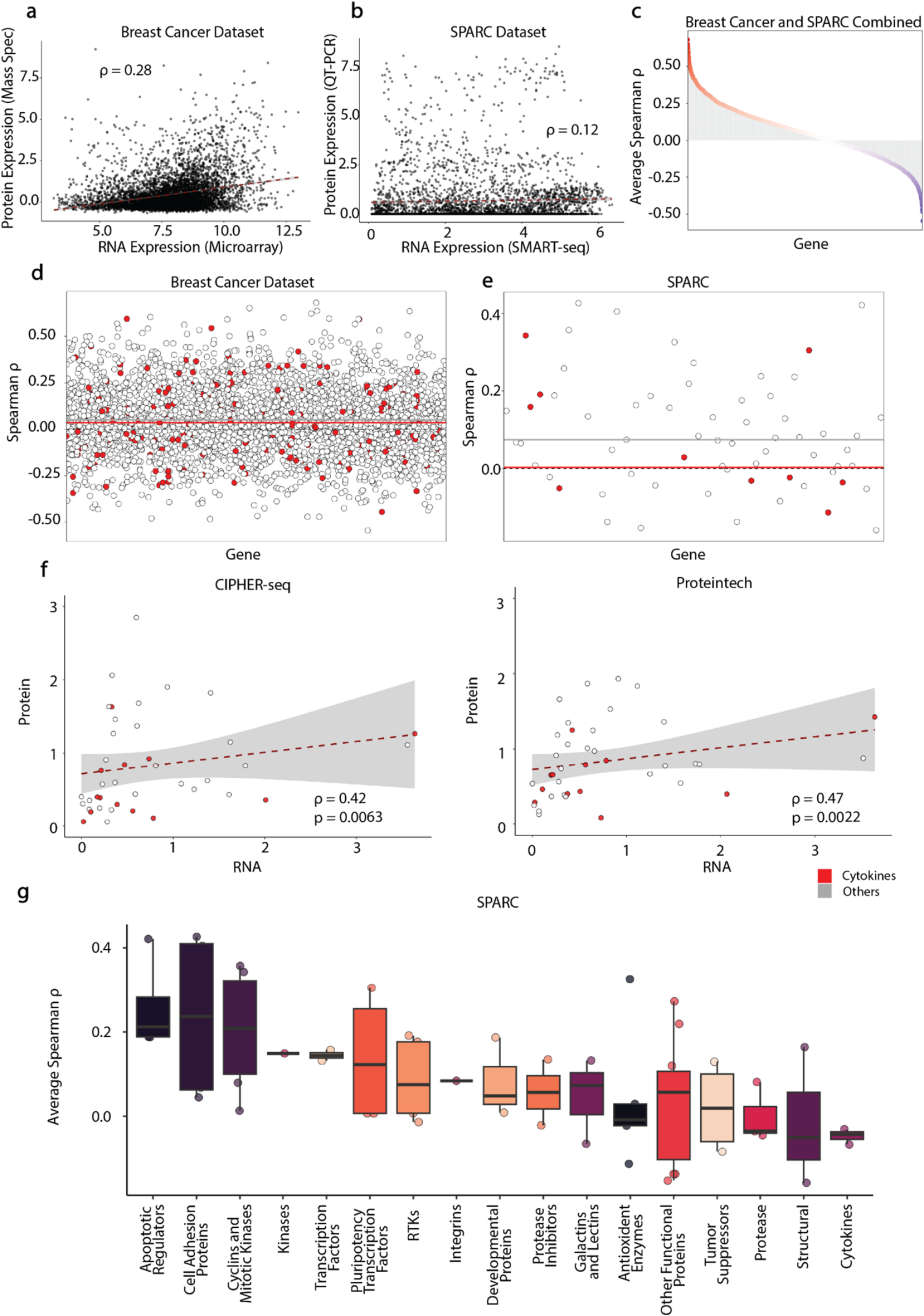
Global RNA-protein discordance in external datasets and intracellular CITE-seq. **(a)** Gene-level RNA vs. protein abundance in the Breast Cancer (healthy and tumor samples) proteogenomic dataset^5^ shows weak concordance (Spearman ρ = 0.28). **(b)** SPARC (48 h timepoint) single-cell RNA-protein measurements^6^ similarly show low correlation (ρ = 0.12). **(c)** Ranked correlations for 3,311 shared genes across both datasets reveal most genes cluster near zero, with many showing negative association. **(d-e)** Gene-wise correlations in healthy Breast Cancer samples (d) and SPARC (e) highlight cytokines and activation markers (red) as consistently among the lowest-correlating features. **(f)** Intracellular CITE-seq scatter plots from PBMCs processed with CIPHER-seq (left) or Proteintech (right) improve RNA-protein concordance (ρ = 0.42 and ρ = 0.47), though cytokines remain discordant. (RNA: mean log-normalized UMI and protein: mean CLR-Normalized ADT) **(g)** Functional-category analysis shows highest concordance for apoptotic and cell adhesion markers and lowest for cytokines, emphasizing that transcript levels poorly predict intracellular protein abundance.

**Fig. 2:**
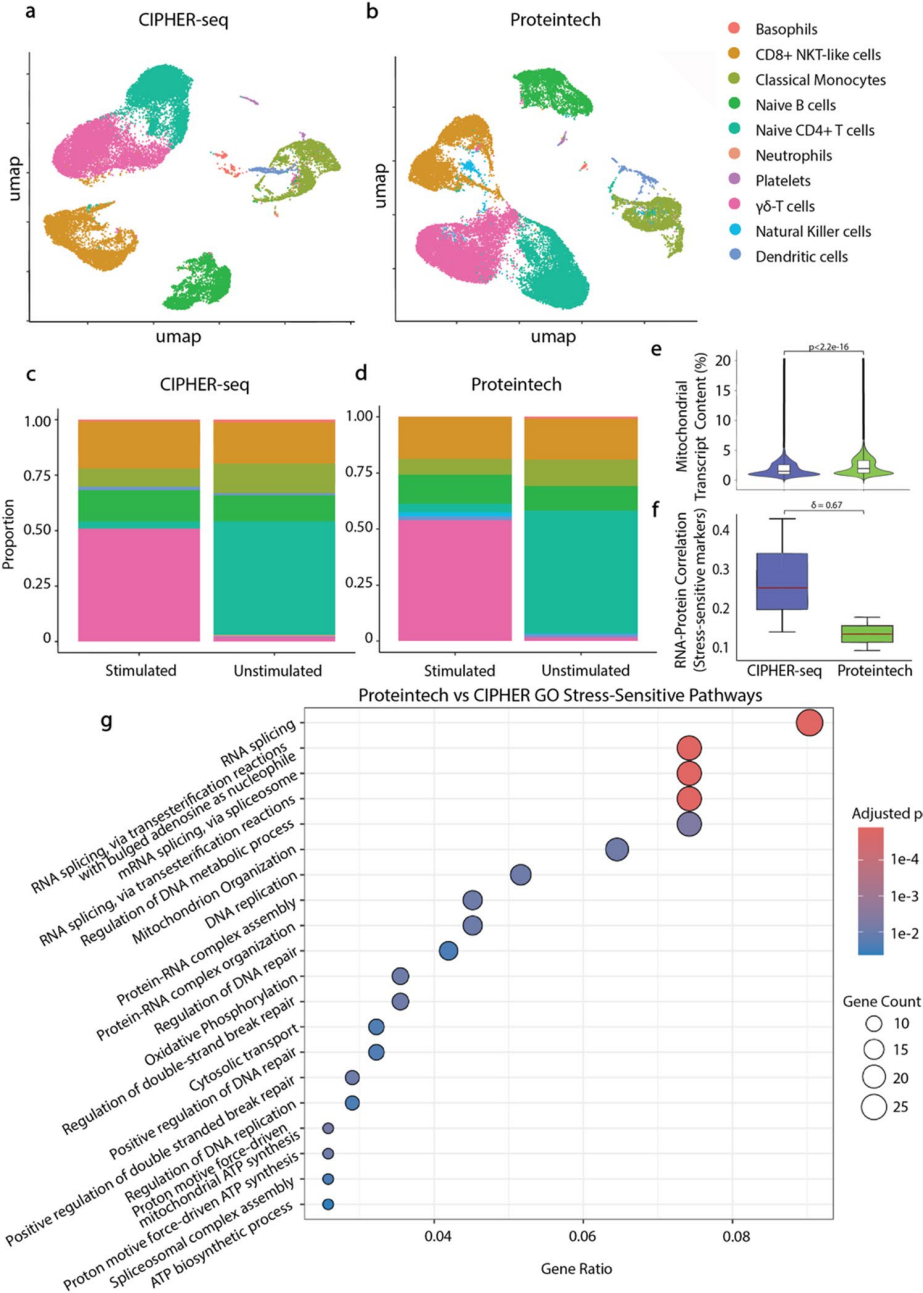
Benchmarking intracellular chemistries highlights reduced stress and improved RNA-protein coupling with CIPHER-seq. **(a-b)** UMAPs of PBMCs processed using CIPHER-seq or Proteintech show similar recovery of major immune lineages by ScType annotation. **(c-d)** Stimulated and unstimulated samples display comparable proportional distributions across cell types for both chemistries. **(e)** Mitochondrial transcript percentages post filtering are significantly higher (Wilcoxon rank-sum test *p* < 2.2e-16) with Proteintech, indicating increased fixation-induced stress. **(f)** Stress-sensitive markers show stronger RNA-protein correlations (Cliff’s delta δ = 0.67) with CIPHER-seq, reflecting reduced artifact-driven stress-sensitive responses. **(g)** GO pathway enrichment identifies pathways where CIPHER-seq better preserves RNA-protein concordance relative to Proteintech.

## Results

We reanalyzed two proteotranscriptomic datasets, Breast Cancer^5^ (GEO accession GSE37751, ProteomeXchange Consortium PXD005692) and SPARC^6^ (SciLifeLab Data Repository (DOI: 10.17044/scilifelab.14207462)), to quantify global RNA-protein concordance. Both cohorts exhibited weak correlations between RNA and protein abundance at the gene level (Fig. 1a-b). Rankings of per-gene Spearman correlation coefficients showed distributions centered near zero, with many genes exhibiting negative correlations (Fig. 1c). Gene-level correlation profiles highlighted cytokines and immune activation genes among the least concordant features across both datasets (Fig. 1d-e). Because RNA and protein in the SPARC and Breast Cancer datasets originate from different technologies and non-matched samples, the resulting correlations may reflect substantial cross-platform/sample noise. Separate regression analyses for cytokine and non-cytokine genes further confirmed reduced RNA-protein concordance among cytokines (Extended Data Fig. 3).

**Fig. 3:**
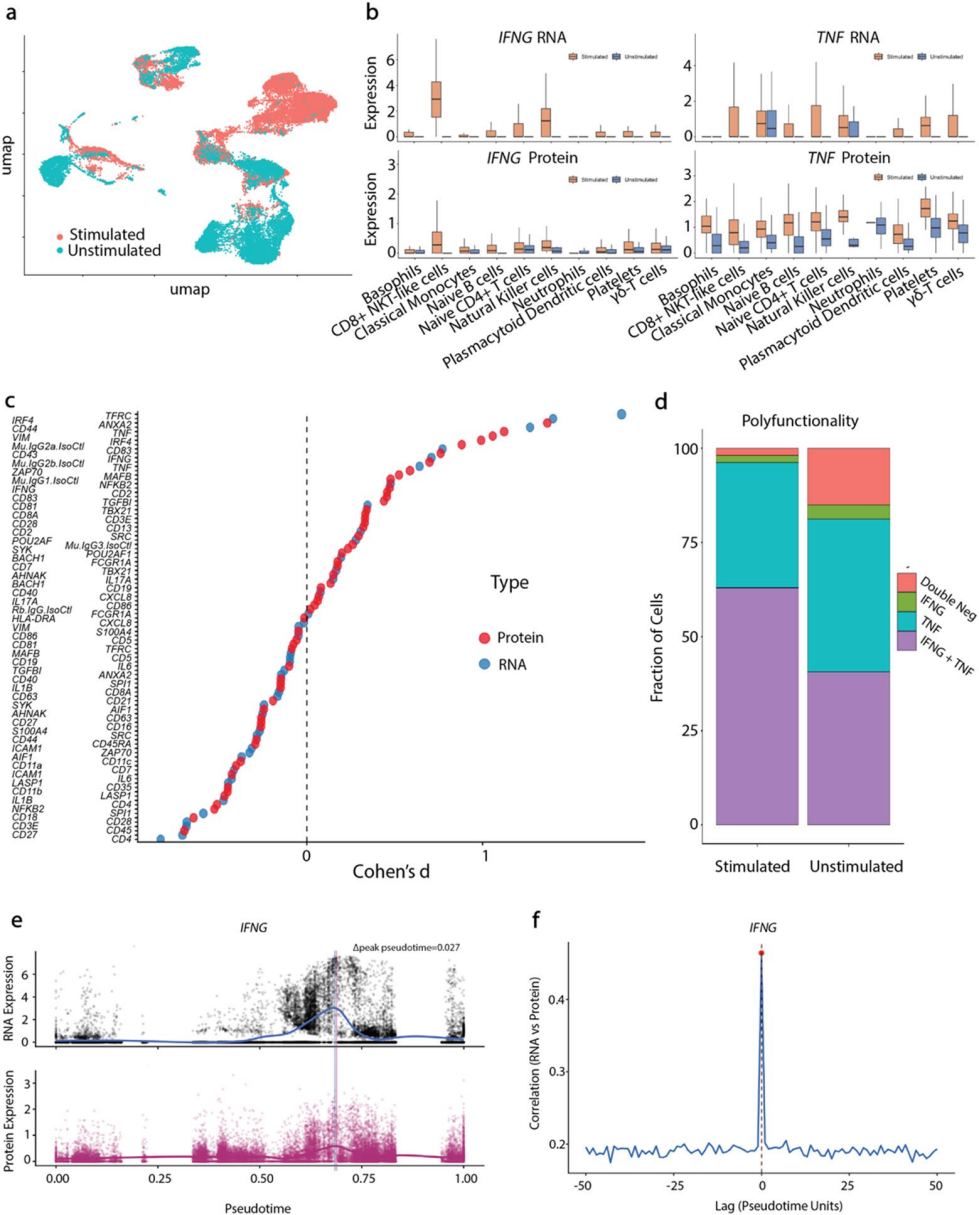
CIPHER-seq captures coordinated RNA and protein dynamics during immune activation. **(a)** UMAP of stimulated vs. unstimulated PBMCs shows activation-driven remodeling. **(b)** *IFNG* and *TNF* RNA and protein levels rise across multiple immune lineages following stimulation. **(c)** Cohen’s d ranking identifies cytokines among the top responders at both RNA and protein levels. (RNA and protein measurements for each feature are plotted at matched positions along the x-axis. Feature names are staggered vertically for readability but correspond to distinct points on the plot.) **(d)** Polyfunctionality analysis shows stimulation-dependent expansion of *IFNG*⁺, *TNF*⁺, and double-positive cells. **(e)** Pseudotime trajectories reveal early *IFNG* RNA induction followed by protein accumulation with a delta value of 0.027. **(f)** Cross-correlation analysis shows a slight positive pseudotime lag, indicating transcription precedes protein expression during activation. The red dashed vertical line indicates the lag corresponding to the maximum cross-correlation value (red dot). Lag values are expressed in pseudotime units.

We next measured RNA-protein correlations using intracellular CITE-seq data generated with CIPHER-seq and Proteintech. Both methods produced modest but improved correlation coefficients relative to external datasets, with CIPHER-seq and Proteintech showing Spearman ρ values in the 0.4–0.5 range (Fig. 1f). While this represents only a moderate correlation with CIPHER-seq (ρ ≈ 0.42), it is important to note that RNA-protein discordance is a well-established biological phenomenon arising from post-transcriptional regulation, differential protein stability, secretion dynamics, and temporal offsets between transcription and translation, processes that are particularly pronounced for cytokines and activation-associated immune genes. Accordingly, even an ideal measurement platform is not expected to yield uniformly high correlations across all gene classes.

Functional stratification of genes into biological categories revealed that apoptosis markers showed the highest average RNA-protein concordance, whereas cytokines remained systematically discordant (Fig. 1g). Time-course related deviations were addressed in the SPARC study^6^. To further quantify this class-specific effect, we performed separate regression analyses for cytokine and non-cytokine genes within each intracellular workflow (Extended Data Fig. 3). In both CIPHER-seq and Proteintech datasets, non-cytokine genes exhibited stronger RNA-protein coupling and steeper regression slopes, whereas cytokines demonstrated reduced correlation coefficients and flatter slopes. In CIPHER-seq, non-cytokines showed a Spearman ρ of 0.45 (*p* = 0.018) compared to 0.31 (*p* = 0.31) for cytokines; a similar pattern was observed with Proteintech (non-cytokines: ρ = 0.42; cytokines: ρ = 0.27). Notably, correlation values varied across features, with the highest gene-level correlation reaching ρ = 0.697 in CIPHER-seq. (Extended Data Fig. 3) Together, these analyses underscore that mRNA abundance alone is an unreliable surrogate for intracellular protein levels, especially for cytokines.

We next benchmarked CIPHER-seq against Proteintech using PBMCs processed in parallel. UMAP embeddings annotated with ScType^14^ demonstrated that both methods recovered comparable repertoires of immune subsets (Fig. 2a-b). Proportional representation of these populations was similar between stimulated and unstimulated conditions for both chemistries (Fig. 2c-d), indicating that neither protocol induced major biases in cell-type recovery. RNA complexity (nFeature_RNA) and total UMI counts showed similar distributions across workflows, with only modest differences in median values (Extended Data Fig. 2e; Extended Data Table 2). These differences do not account for the substantially higher mitochondrial transcript enrichment observed with Proteintech, supporting the interpretation that this is a stress-associated response.

Despite similar cellular composition, key RNA quality metrics diverged markedly between the protocols. Proteintech-processed samples exhibited substantially elevated mitochondrial transcript percentages across cell types, consistent with fixation-induced cellular stress and compromised RNA integrity, whereas CIPHER-seq samples maintained low mitochondrial content (Fig. 2e). RNA complexity and total UMI counts remained comparable, indicating that the principal difference was a shift toward stress-associated mitochondrial reads rather than changes in captured molecules.

To assess whether fixation influenced RNA-protein concordance among stress-sensitive genes represented in our panel, we examined apoptosis-associated protein-RNA. CIPHER-seq maintained significantly higher RNA-protein concordance for these genes relative to Proteintech based on Cliff’s delta values (Fig. 2f, Extended Data Table 3), suggesting that Proteintech’s fixation conditions introduce artifactual stress programs that decouple transcripts from protein abundance. Gene ontology analysis on pseudobulk RNA for both samples showed enhanced correlation under CIPHER-seq, highlighting mitochondrial organization, ATP synthesis, oxidative phosphorylation, DNA replication and repair, and RNA splicing (Fig. 2g). These processes represent stress-sensitive cellular and metabolic processes susceptible to perturbation during cellular activation and fixation^12–16^.

Collectively, these data demonstrate that CIPHER-seq retains high-quality transcriptomes and preserves biologically meaningful RNA-protein relationships while matching Proteintech in immune cell recovery. Reduced mitochondrial stress and enhanced correlation within metabolic and stress pathways highlight CIPHER-seq as a less perturbing intracellular chemistry for multiomic profiling.

We next applied CIPHER-seq to stimulated PBMCs to quantify intracellular cytokine responses. Stimulation induced strong, coordinated *IFNG* and *TNF* upregulation across immune subsets (Fig. 3b), with cytokines among the features showing the largest effect sizes (Fig. 3c) and an expansion of *IFNG*⁺, *TNF*⁺, and double-positive polyfunctional cells (Fig. 3d).

Pseudotime analysis revealed that *IFNG* RNA induction preceded peak protein accumulation, with a measurable Δpeak pseudotime of 0.027 (Fig. 3e), indicating that maximal RNA expression occurred earlier along the inferred activation trajectory. Although the RNA and protein peaks were closely spaced, they were not coincident, consistent with a short temporal offset between transcription and intracellular protein accumulation.

Cross-correlation analysis further demonstrated that maximal RNA-protein concordance occurred near zero lag with a slight shift favoring RNA leading protein (Fig. 3f). The small magnitude of this lag suggests near-synchronous dynamics within the resolution limits of pseudotime inference, while still supporting the expected biological ordering of transcription preceding protein accumulation. Together, these analyses indicate that RNA and protein measurements capture closely coupled but not identical activation kinetics, reinforcing the value of simultaneous multimodal profiling.

Global UMAP structure separated stimulated from unstimulated states (Extended Data Fig. 4a) with increased mitochondrial and transcriptional activity (Extended Data Fig. 4b). Cytokine responses varied by lineage (Extended Data Fig. 4c-e), consistent with known variation in activation thresholds^17,18^. Coordinated increases in *IFNG* and *TNF* RNA and protein across subsets further validated multimodal activation consistency (Extended Data Fig. 4f).

**Fig. 4:**
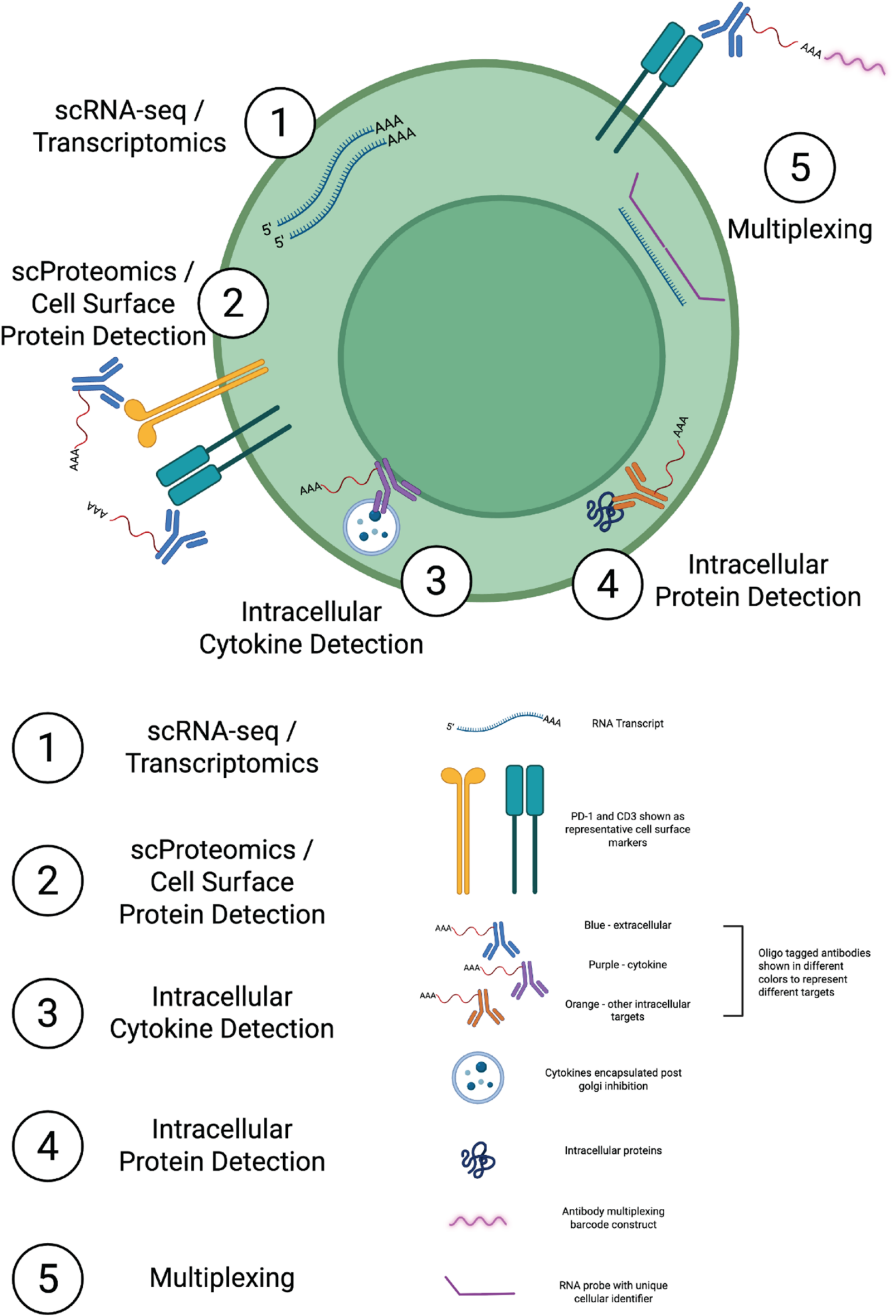
Five-layer multimodal architecture of CIPHER-seq. Schematic representation of the five information layers captured by CIPHER-seq: (1) whole-transcriptome scRNA-seq, (2) cell-surface protein profiling, (3) intracellular cytokine detection, (4) intracellular protein measurement, and (5) sample multiplexing via oligo-tagged barcodes and unique RNA probes. Distinct color-coded antibodies illustrate simultaneous detection of extracellular markers, cytokines, and other intracellular proteins within the same cell. Created in BioRender (Bhalgat, A. (2025) https://BioRender.com/a93ma9q).

## Conclusion

These findings establish CIPHER-seq as a robust platform for intracellular multimodal profiling (Fig. 4) that overcomes key limitations of existing protocols. By minimizing fixation-induced stress while enabling sensitive detection of intracellular cytokines, CIPHER-seq generates high-quality, interpretable data that capture both the magnitude and timing of immune responses.

## Methods

### Human PBMC acquisition and handling

Fresh human peripheral blood mononuclear cells (PBMCs) were purchased from StemCell Technologies (Human Peripheral Blood Mononuclear Cells, Fresh, 100 million; Cat. 200 − 0077). Units were shipped overnight at 4 °C and processed immediately upon arrival. Cells were transferred to 50-mL conical tubes, diluted at least 1:1 with room-temperature PBS supplemented with 0.04% bovine serum albumin (BSA), and centrifuged at 300 × g for 10 min. Supernatants were discarded and pellets gently resuspended in PBS + 0.04% BSA. Cell counts and viability were measured using trypan blue and a Countess II FL automated cell counter. Only preparations with viability ≥ 95% were used. For all downstream steps, cells were kept on ice or at 4 °C whenever possible to minimize transcriptional changes prior to fixation.

PBMCs used for single-cell sequencing-based benchmarking of CIPHER-seq and the Proteintech workflow were derived from a single healthy donor. These experiments were conducted to evaluate technical performance, including RNA integrity, mitochondrial transcript enrichment, and RNA-protein coordination, rather than to assess inter-individual biological variability.

Additional PBMC donors were used during protocol development and optimization stages, including intracellular flow cytometry validation and RNA quality surrogate endpoint analyses across multiple fixation and permeabilization conditions; however, these experiments were not advanced to full single-cell sequencing.

### Stimulation conditions

PBMCs were resuspended at 1 × 10⁶ cells/mL in complete RPMI-1640 (10% heat-inactivated fetal bovine serum, 1% penicillin/streptomycin, 2 mM L-glutamine). For stimulated conditions, cells were treated with phorbol 12-myristate 13-acetate (PMA, 50 ng/mL) and ionomycin (1 µg/mL) for 4 h at 37 °C, 5% CO₂. Brefeldin A (5 µg/mL) was added during the final 3 h to block protein secretion and retain cytokines intracellularly. Unstimulated controls were incubated in parallel in the same medium without PMA/ionomycin but with brefeldin A added for the final 3 h to match handling. At the end of incubation, cells were washed twice in cold PBS + 0.04% BSA and counted before fixation.

### CIPHER-seq fixation, permeabilization, and intracellular antibody labeling

CIPHER-seq is built around a modified Cytokine Flow Cytometry^19^ workflow designed to preserve RNA while allowing access to intracellular proteins, representing a method-level innovation. Key optimization allowed for compatibility with scRNAseq techniques. (Extended Data Fig. 1)

**Fixation**: Washed cells were resuspended at 1–5 × 10⁶ cells/mL in 50ul Caltag Fix & Perm (Nordic MuBio, Cat. GAS-002) Reagent A supplemented with RNase inhibitor and incubated for 20 min at room temperature. After fixation, cells were washed twice with Staining Buffer (0.1% sodium azide and 1% FBS in PBS) to remove excess fixative. **Blocking and RNase protection**: Fixed cells were resuspended in blocking buffer (Staining buffer supplemented with 5ul of Human TruStain FcX and RNase inhibitor) and incubated on ice for 10 min to reduce nonspecific antibody binding and protect RNA. **Permeabilization and intracellular staining**: Cells were pelleted and resuspended in Caltag Fix & Perm Reagent B supplemented with RNase inhibitor (1 U/µL). Oligonucleotide-conjugated antibodies from the Proteintech Multipro Human Fixed-Cell Immune Profiling Antibody Cocktail were added directly to the permeabilization mix at manufacturer-recommended dilutions. Samples were incubated for 30 min at 4 °C to permit intracellular antibody access. **Post-stain washes**: Following staining, cells were washed twice with Staining Buffer to remove unbound antibodies and residual permeabilization reagent. Final pellets were resuspended at the concentration recommended for 10x Genomics Flex loading.

Conditions for fixation and permeabilization agent exposure time (10–30 min), temperature (RT vs. on ice/4°C), and RNase inhibitor concentration (1–5 U/µL) were optimized empirically to minimize mitochondrial stress signatures and preserve RNA integrity, as assessed by surrogate endpoints.

### Proteintech intracellular protocol

For comparison, the Proteintech Multipro intracellular CITE-seq protocol (Human Discovery Panel, Rev B) was used according to the manufacturer’s online instructions. Briefly, cells were fixed in paraformaldehyde-based fixative, subjected to detergent-based permeabilization, and then stained with the Proteintech oligo-conjugated antibody cocktail. Fixation and permeabilization times and temperatures were those recommended by Proteintech. After intracellular staining, cells were washed and resuspended in PBS + 0.04% BSA prior to 10x Flex processing.

### BD and BioLegend intracellular protocols for flow cytometry benchmarking

BD and BioLegend intracellular chemistries were evaluated by conventional flow cytometry to assess intracellular antigen accessibility. For BD, we used the BD Omics Guard fixation reagent followed by BD IC-Perm Buffer (methanol-free) according to the BD ICOS/ICS Intracellular Staining Protocol. For BioLegend, we used the TotalSeq-B/C Cell Surface and Intracellular Protein Staining protocol, which incorporates BioLegend Fixation Buffer and Intracellular Staining Permeabilization Wash Buffer together with TruStain FcX and monocyte blockers. In both workflows, fluorescently labeled antibodies against *CD3*,* CD23*, and *Tubulin* were used to quantify intracellular staining by flow cytometry (Extended Data Fig. 2). Only Proteintech and CIPHER-seq conditions provided robust *Tubulin* staining, so BD and BioLegend protocols were not advanced to Single-cell sequencing.

### 10x Genomics GEM-X Flex library construction

Cells processed with either CIPHER-seq or Proteintech were loaded onto a Chromium X instrument using the 10x Genomics GEM-X Flex Gene Expression Reagent Kits for Multiplexed Samples with Feature Barcode technology for Protein using Barcode Oligo Capture (CG000789, Rev B). Cell concentrations were adjusted such that targeted cell recovery per channel was 80,000 cells, 20,000 cells per sample.

The workflow included whole-transcriptome probe hybridization using unique probe barcodes. At the same time, antibody multiplexing barcodes were incorporated into the labeled and fixed samples. After the hybridization process, the samples were pooled, washed, and counted using the Nexcelom K2 digital cell counter. Next, Gel Bead-in-emulsion (GEM) was generated by combining the barcoded gel beads with a master mix containing the pooled cells and partitioning oil on the GEM-X FX chip. Following GEM recovery and pre-amplification, gene and protein (antibody-derived tags - ADT library) expression libraries, were constructed according to the 10x Genomics guidelines and sequenced on an Illumina NovaSeq X using paired-end 2 × 151-bp reads. The gene expression and ADT libraries were balanced in the pool to ensure sufficient depth for both modalities.

### Flow cytometry acquisition and analysis

Samples for flow cytometry were acquired on a BD LSRFortessa. Compensation was performed using single-stained compensation beads. Gating strategies began with FSC/SSC to exclude debris, followed by doublet discrimination (FSC-A vs. FSC-H) and live/dead discrimination if applicable. *CD3*⁺ lymphocytes were gated to quantify T-cell staining across protocols. For intracellular targets such as *Tubulin*, gates were set using unstained and single-stained controls. Data were analyzed with FlowJo, and summary statistics for positive populations were exported for bar-graph and heatmap visualization.

### Pre-processing of Single-cell data

Raw sequencing data were demultiplexed with bcl2fastq and processed using Cell Ranger v7.0. Gene-expression matrices and ADT count tables were imported into R (v4.3.1) and analyzed using Seurat v5. Cells were excluded if they had < 200 detected genes, > 8,000 detected genes, or > 20% mitochondrial RNA^20–23^. For some analyses focusing on stress signatures, more stringent mitochondrial cutoffs (e.g. <15% or < 10%) were explored as sensitivity analyses; conclusions were unchanged and therefore the above-reported exclusions have been broadly applied to the data presented here.

Gene-expression counts were normalized and variance-stabilized with SCTransform or log-normalization for gene expression counts. Dimensionality reduction used principal component analysis (PCA), and the first 20 PCs were used to construct a shared nearest-neighbor graph. UMAP embeddings were generated using default parameters. Clusters were identified with the Louvain algorithm at resolutions optimized to separate major immune lineages.

### Cell type annotation

Cell types were assigned using ScType^14^ with the “Immune system” reference and default settings. Automated labels were manually curated based on canonical marker expression patterns (e.g., *MS4A1* and *CD79A* for B cells; *NKG7* and *GNLY* for NK cells; *LST1* and *S100A8* for monocytes; *CCR7* and *IL7R* for naïve CD4 T cells). Annotations were harmonized across CIPHER-seq and Proteintech samples to facilitate direct comparison.

### ADT normalization and cytokine quantification

ADT counts were CLR-normalized across cells within each sample. For visualization, normalized values were centered and scaled by feature. Cytokine-positive cells were defined by thresholding normalized ADT intensities based on isotype or unstimulated controls. Fractions of *TNF*⁺ and *IFNγ*⁺ cells were calculated within each annotated cell type for stimulated and unstimulated conditions.

### RNA-protein correlation analyses

For each gene with both RNA and ADT measurements, Spearman’s correlation coefficients (ρ) were calculated across single cells. Pathway-level analyses were performed by grouping genes into functional categories (e.g., apoptosis, cell cycle, cytokines, metabolism, DNA repair, oxidative phosphorylation, RNA processing) based on curated gene lists from previous studies^5,6,12–16^. Average ρ per pathway was computed and compared between datasets and protocols.

### Differential expression and log2 fold-change

Differential gene expression between stimulated and unstimulated conditions was assessed within each cell type using the Wilcoxon rank-sum test implemented in Seurat. P values were adjusted using Bonferroni correction. Log2 fold changes between stimulated and unstimulated cells were computed for cytokine genes and summarized as heatmaps. For ADT data, analogous tests were performed on CLR-normalized counts.

### Pseudotime, effect size, and polyfunctionality analyses

To examine temporal relationships between RNA and protein expression, cells were ordered along an activation trajectory using a graph-based pseudotime algorithm implemented in R. The trajectory was anchored in unstimulated cells and progressed toward highly stimulated states based on global transcriptional changes. For *IFNG*, smoothed expression curves for RNA and protein were plotted along pseudotime. To quantify timing differences, cross-correlation between RNA and protein profiles was computed across pseudotime lags; the lag with maximum correlation was reported.

Effect sizes for stimulation (Cohen’s d) were calculated for each cytokine at both RNA and protein levels by comparing stimulated and unstimulated cells within each cell type. Polyfunctionality was quantified by classifying cells as double-negative, *IFNG*-only, *TNF*-only, or *IFNG*⁺*TNF*⁺ and visualizing fractions in stimulated versus unstimulated samples.

### External dataset processing

Breast cancer proteotranscriptomic data^5^ and SPARC Single-cell data^6^ were downloaded from the authors’ repositories.

The breast cancer proteogenomic dataset was accessed using transcriptomic data from the Gene Expression Omnibus (GEO accession GSE37751) and corresponding mass spectrometry-based proteomic data from the ProteomeXchange Consortium (dataset identifier PXD005692).

The SPARC single-cell proteotranscriptomic dataset was obtained from the SciLifeLab Data Repository (DOI: 10.17044/scilifelab.14207462), which provides processed single-cell RNA and intracellular protein measurements as described in Reimegård et al.

RNA and protein matrices were log-normalized as in the original publications. Gene-wise Spearman correlations were computed and ranked to generate distributions shown in Fig. 1a-e, g. Functional categories were mapped using the same gene sets as used for our internal analyses.

### Statistical analysis

All statistical tests were two-sided unless otherwise noted. *P* < 0.05 was considered significant. Violin plots display kernel density estimates with embedded boxplots showing median and interquartile ranges. Boxplots depict medians, interquartile ranges, and whiskers extending to 1.5x the interquartile range. Heatmaps reflect row-wise z-scoring unless stated otherwise. Analyses were performed in R v4.3.1 using Seurat v5, tidyverse, and ComplexHeatmap packages.

For comparisons between two groups (e.g., CIPHER-seq vs. Proteintech), two-sided Wilcoxon rank-sum tests were used unless otherwise specified. Exact p-values are reported in the corresponding figures and figure legends.

Effect sizes were quantified using Cliff’s delta (δ), a non-parametric measure of effect size appropriate for distributions that may deviate from normality. Cliff’s delta values range from − 1 to 1, with values closer to ± 1 indicating stronger effects. For interpretative context, |δ| < 0.147 was considered negligible, 0.147–0.33 small, 0.33–0.474 moderate, and > 0.474 large, consistent with established guidelines^24^.

### Figure Generation

Figures were generated using FlowJo v10.10.1, Rstudio v2025.09.2 + 418, R v4.3.1, and BioRender (biorender.com; accessed 2025).

## Supplementary Information

Below is the link to the electronic supplementary material.

Supplementary Material 1

## Data Availability

The datasets generated and/or analyzed during the current study are available in the Gene Expression Omnibus repository, GSE314400.
