# Supplementary material for: CIPHER-seq enables intracellular multimodal profiling of cytokine responses in single immune cells
Source: Sci Rep. 2026 Apr 8;16:9693. doi: 10.1038/s41598-026-44946-y (PMC13061944; doi:10.1038/s41598-026-44946-y)
Supplement: Supplementary file 1 — Supplementary Material 1 [file 41598_2026_44946_MOESM1_ESM.pdf]

Extended Data

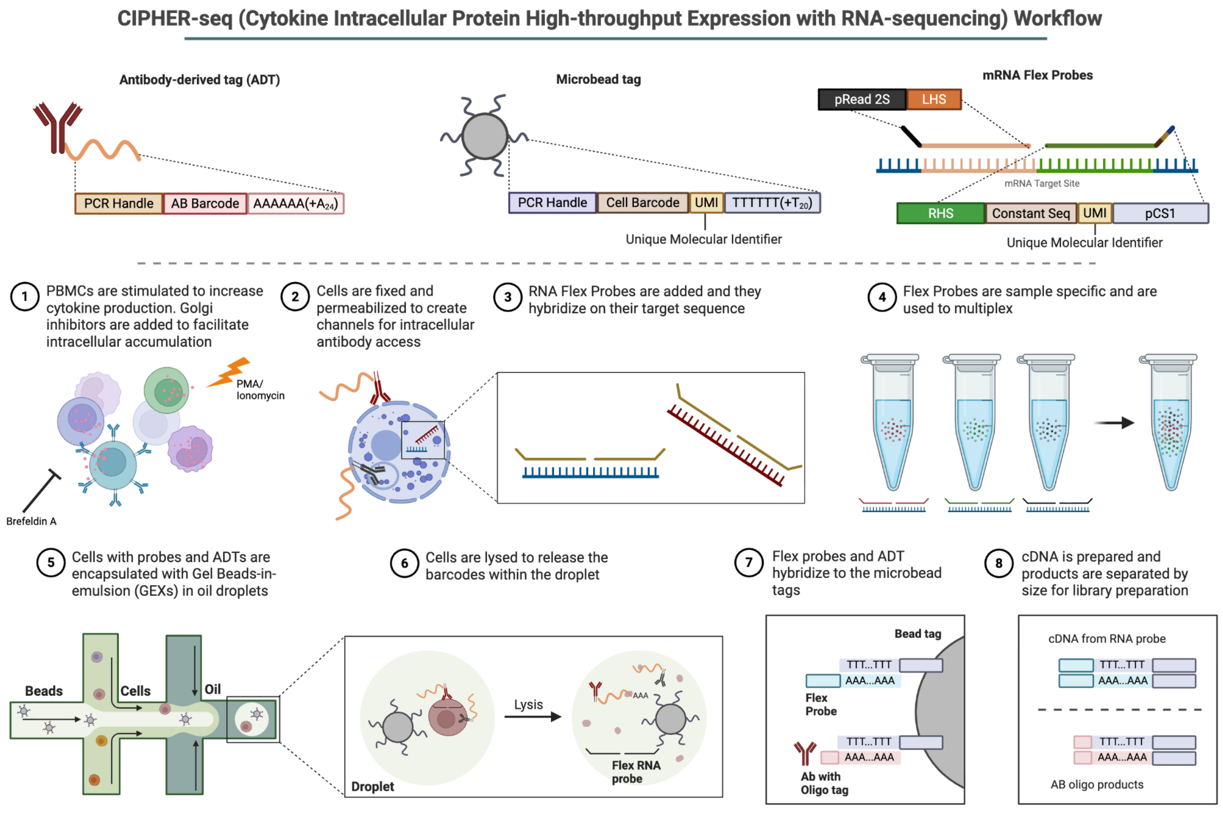

Extended Data Figure 1. Overview of the CIPHER-seq workflow.

Stepwise schematic illustrating the CIPHER-seq chemistry. PBMCs undergo PMA/ionomycin stimulation in the presence of Golgi inhibitors to enable intracellular cytokine accumulation. Cells are then fixed with Caltag Fix & Perm Reagent A and permeabilized with Reagent B, allowing entry of oligo-tagged antibodies. RNA Flex probes hybridize to target transcripts prior to encapsulation with barcoded gel beads in emulsion droplets. Upon lysis, intracellular protein tags and RNA probes bind to bead-linked barcodes, after which cDNA and ADT fragments are size-separated and prepared for 10x Genomics Flex library construction. Created in BioRender. (Bhalgat, A. (2026) https://BioRender.com/q34m8bs)

Metrics	BD	BioLegend	Proteintech	CIPHER-seq
**Cost per Sample**	$30-$50	$25-$40	$20-$30	$10-$20
**Protocol**				
Fixation Agent	BD Omics Guard	BioLegend Fixation Buffer	4% Paraformaldehyde	Caltag Fix Perm Agent A
Permeabilization Method	BD IC-Perm Buffer (methanol based)	Intracellular Staining Permeabilization Wash Buffer	Tween-20 + NP-40	Caltag Fix Perm Agent B
Special Reagents	Fc Block and AbSeq Enhancer	TruStain FcX Plus, True-Stain Monocyte Blocker	Enhanced Blocker	None
Overall Protocol Time	~6 hours (overnight option)	~4.5 hours (overnight option)	~3 hours	~3 hours
**Advantages**	Overnight stop step	Overnight stop step	Quick protocol	Quick protocol, cheapest, mostly done at RT, most shelf stable reagents
**Disadvantages**	Most expensive, requires the Rhapsody system for sequencing	Longer protocol, Specialized reagents	Need for specialized reagents, no overnight stop step	No commercial support

Extended Data Table 1. Comparison of intracellular CITE-seq protocols.

Summary of workflow characteristics, chemical compositions, and performance features for BD, BioLegend, Proteintech, and CIPHER-seq intracellular staining protocols. Metrics include cost per sample, fixation chemistry, permeabilization strategy, specialized reagents, total protocol time, and qualitative advantages and disadvantages. CIPHER-seq is the fastest and most cost-effective protocol, avoids overnight incubations, and requires no specialized blockers or enhancers.

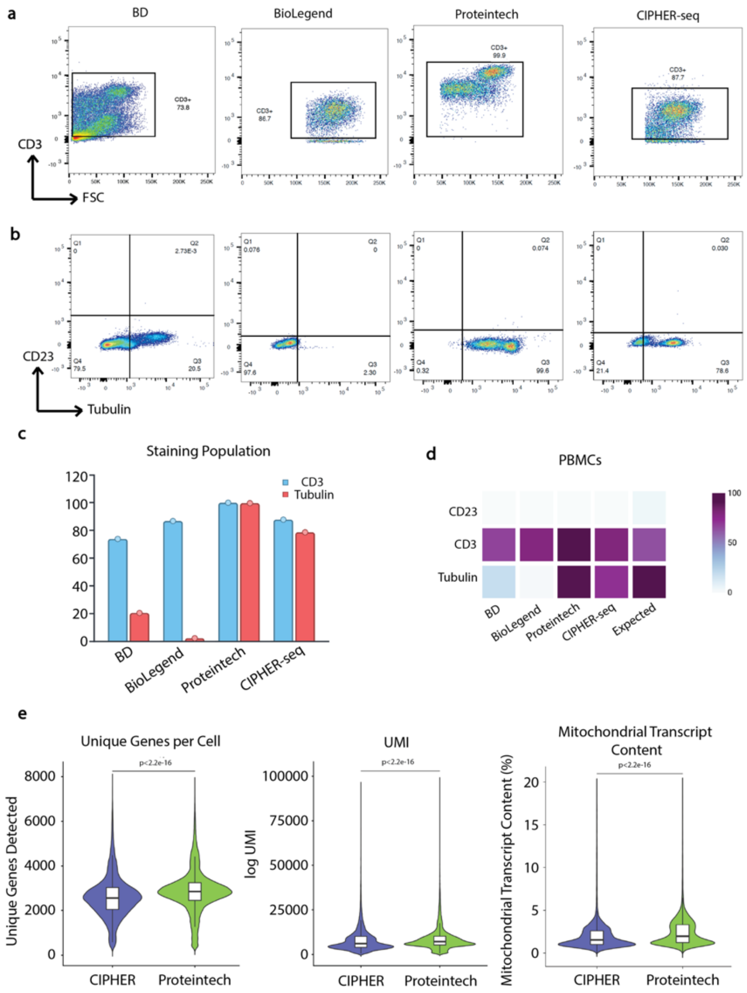

Extended Data Figure 2. Flow cytometric benchmarking of intracellular staining protocols in healthy donor PBMCs.

All samples were run and acquired simultaneously, but each method yielded very different scatter and fluorescence patterns. Gating was benchmarked on unstained, fix/perm samples for each method to account for physiological variability. **(a)** CD3 staining across BD, BioLegend, Proteintech, and CIPHER-seq intracellular workflows shows variable detection of surface markers following fixation. **(b)** Representative CD23 versus Tubulin intracellular staining demonstrates that BD and BioLegend chemistries fail to expose cytoskeletal epitopes, whereas Proteintech and CIPHER-seq enable robust Tubulin detection. **(c)** Quantification of CD3 and Tubulin staining percentages across protocols shows uniformly low intracellular target detection with BD and BioLegend, moderate performance with Proteintech, and high performance with CIPHER-seq. **(d)** Heatmap summarizing observed detection frequencies for CD23 (negative extracellular control), CD3 (positive extracellular control), and Tubulin across protocols relative to expected expression patterns in PBMCs, highlighting differences in intracellular antigen accessibility prior to single-cell multiomic profiling. **(e)** RNA quality metrics following single-cell sequencing, including unique genes per cell, total UMI counts, and mitochondrial transcript percentage, comparing CIPHER-seq and Proteintech workflows.

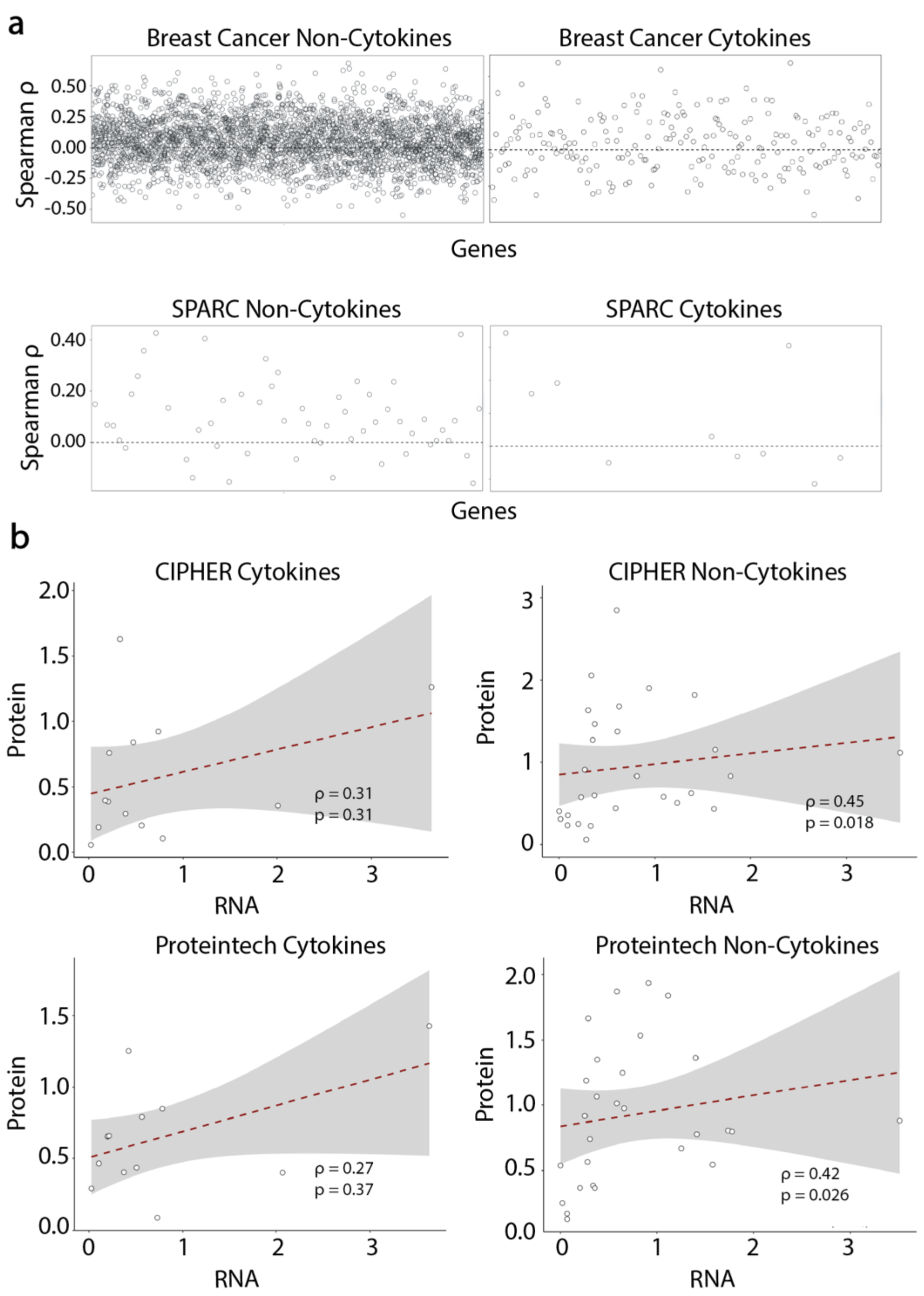

Extended Data Figure 3. Gene-class specific RNA-protein concordance across datasets and workflows.

(a) Gene-level Spearman correlation coefficients (ρ) for non-cytokine and cytokine genes in external breast cancer and SPARC datasets. Cytokines display lower RNA-protein concordance relative to non-cytokine genes across platforms. **(b)** Separate regression analyses for cytokine and non-cytokine gene-protein pairs measured by CIPHER-seq and Proteintech. Scatterplots show mean log-normalized RNA expression versus mean CLR-normalized ADT protein expression with linear regression fits (red dashed lines) and 95% confidence intervals (shaded). Spearman correlation coefficients (ρ) and p-values are indicated in each panel. Non-cytokine features exhibit higher concordance than cytokines in both workflows.

Metric	CIPHER (Median)	CIPHER (Q1)	CIPHER (Q3)	Proteintech (Median)	Proteintech (Q1)	Proteintech (Q3)
**Mitochondrial transcript (%)**	1.52	1.00	2.58	1.95	1.22	3.31
**Total UMI counts**	6,071	3,987	10,085	7,186	5,323	10,193
**Unique genes detected (nFeature)**	2,550	2,044	3,018	2,839	2,449	3,236

Extended Data Table 2. Summary statistics of single-cell RNA quality metrics across workflows.
Median and interquartile range (IQR) values are shown for mitochondrial transcript percentage, total UMI counts, and number of detected genes (nFeature) per cell following quality control filtering (mitochondrial transcript percentage <20%). Metrics are reported for CIPHER-seq and Proteintech workflows to provide quantitative context for comparisons of RNA complexity and stress-associated signatures.

Gene Symbol (RNA)	ADT Target Name	Functional Annotation
TNF	TNF.1	Pro-inflammatory cytokine; apoptosis signaling
IL1B	IL1B.1	Inflammatory mediator; cell death signaling
NFKB2	NFKB2.1	NF-κB pathway; survival/apoptosis regulation
TGFBI	TGFBI.1	TGF-β signaling; extracellular matrix remodeling
AIF1	AIF1.1	Apoptosis-inducing factor

Extended Data Table 3. RNA-protein feature pairs included in stress-sensitive pathway analysis.
List of gene symbols (RNA), corresponding ADT targets (protein), and functional annotations for markers evaluated in the CIPHER-seq workflow. This table includes all protein-gene pairs that were assessed for RNA-protein concordance and stress-sensitive pathway analyses shown in Fig. 2.

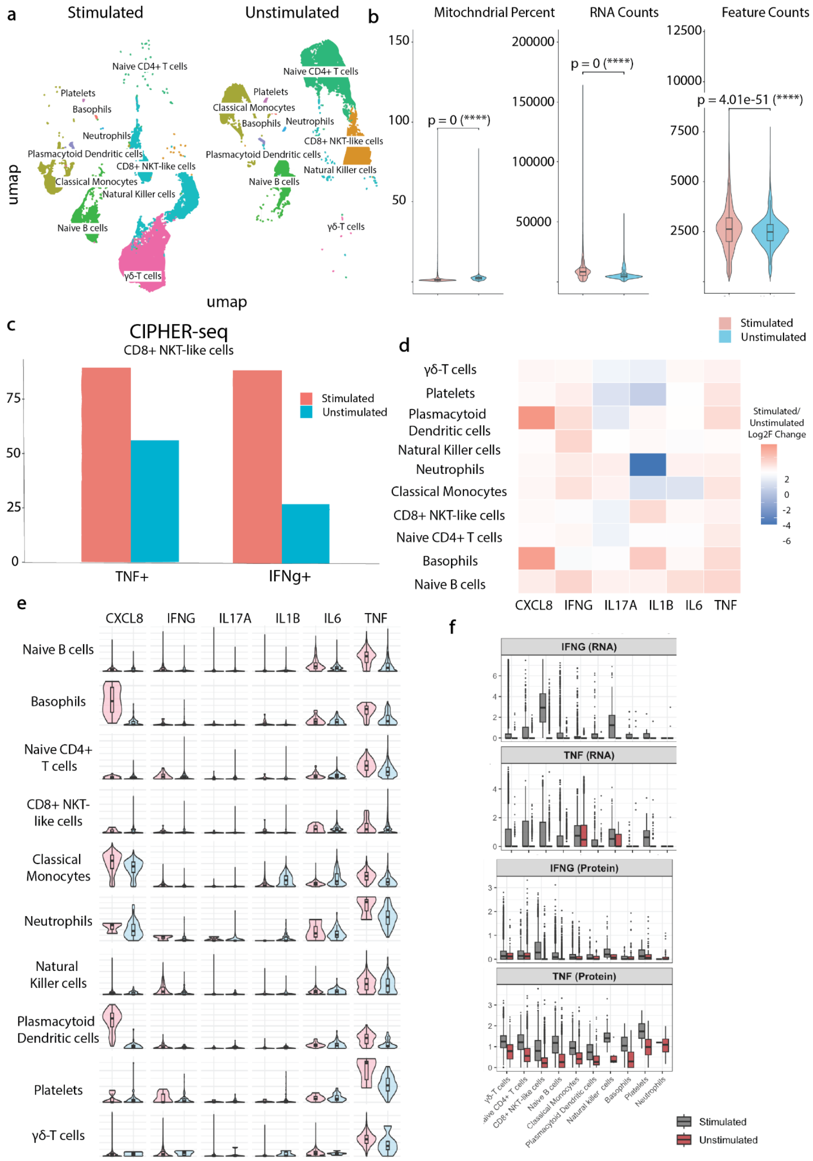

Extended Data Figure 4. CIPHER-seq captures cell-type-specific cytokine induction and metabolic activation in stimulated PBMCs.

(a) UMAP visualization of stimulated and unstimulated PBMCs processed with CIPHER-seq, demonstrating global transcriptional remodeling following PMA/ionomycin activation. **(b)** Violin plots of mitochondrial percentage, RNA counts, and number of detected genes, reflecting increased metabolic activity and transcriptional output in stimulated cells. **(c)** Intracellular induction of TNF and IFNG within $CD8^+$ NKT-like cells, highlighting robust activation. **(d)** Cytokine log_2 fold-change heatmap showing strong induction of CXCL8, IL1B, and TNF in plasmacytoid dendritic cells and basophils, with suppression of IL1B in neutrophils. **(e)** Violin plots of CXCL8, IFNG, IL17A, IL1B, IL6, and TNF RNA across immune lineages demonstrating heterogeneous cytokine responsiveness. **(f)** Boxplots comparing IFNG and TNF RNA and protein across cell types, showing coordinated induction with cell-type-specific magnitudes.
